# Household catastrophic health expenditures for rheumatoid arthritis: a single centre study from South India

**DOI:** 10.1038/s41598-023-42623-y

**Published:** 2023-09-16

**Authors:** Bhavani Shankara Bagepally, S. Sajith Kumar, Akhil Sasidharan, Madhumitha Haridoss, Krishnamurthy Venkataraman

**Affiliations:** 1 National Institute of Epidemiology (ICMR), Health Technology Assessment Resource Centre ICMR-NIE, R-127, Tamil Nadu Housing Board, Phase I and II, Ayapakkam, Chennai, 600077 India; 2 Chennai Meenakshi Multispecialty Hospital, Chennai, India

**Keywords:** Osteoimmunology, Health care economics, Health policy, Health services

## Abstract

Rheumatoid arthritis (RA) not only has a physical and emotional toll but also has a substantial economic impact. This study aims to estimate the burden of catastrophic health expenditure (CHE) on households due to RA in Tamil Nadu, India. We conducted cross-sectional descriptive hospital-based single-centre study at a tertiary care private multispecialty hospital in Tamil Nadu, India. The study comprised 320 RA patients who visited the outpatient clinic from April to October 2022. Demographic and baseline descriptive characteristics were reported. Multivariable logistic regression analyses were performed to identify major determinants associated with CHE. We also examined the inequality in household annual income and CHE. Most study participants were females (88.1%) with a mean age (SD) of 55.57 ± 12.29 years. About 93% of RA patients were from urban areas, and 89.4% were literate. Only 8.1% of respondents reported having health insurance. Households experiencing CHE owing to RA were 51.4% (n = 162). The mean (95% CI) annual health expenditure for treating RA is ₹44,700 (₹41,710 to 47,690) with a median (IQR) of ₹39,210 (₹25,500) [$476 ($310)]. The corresponding mean (95% CI) and median (IQR) Out of pocket expenditure among RA patients per household were ₹40,698 (₹38,249 to 43,148) [$494 ($464 to $524)] and ₹36,450 (23,070) [$442 ($280)] respectively. Nearly half of the households with RA patients had a financial catastrophe due to healthcare costs being paid out-of-pocket and limited health insurance coverage. The results underscore the need for comprehensive approaches to strengthening public health policies along with financial risk protection and quality care in India.

## Introduction

Rheumatoid arthritis (RA) is a chronic autoimmune disease causing inflammation, pain, and stiffness that afflicts women up to five times more than men^1^. It causes significant morbidity and mortality, affecting 1% of the world population^2^. In India, the prevalence of RA is estimated to be 0.7%^3^ which is higher than the global prevalence of 0.46%^4^. Most of RA patients suffer long-lasting illnesses, which significantly reduce their levels of physical activity and negatively impact their quality of life^5^. The treatment of RA has evolved over the last few decades, particularly since the advent of biological/targeted disease-modifying antirheumatic drugs (DMARDs)^6^. Conventional synthetic disease-modifying anti-rheumatic drugs (csDMARDs) are prescribed as the first-line treatment for RA according to the standard treatment guidelines^7,8^. With csDMARDs failure, newer treatments such as biological/targeted DMARDs are recommended^9^.

RA treatment is expensive, particularly with biologics/targeted therapies, which has a significant economic impact^9^. The healthcare system in India is characterized by a mix of public and private providers. The majority of Indians seek treatment from the private sector, where over two-thirds of overall health spending is through out-of-pocket^10^. The high cost of care and a lack of health insurance coverage exacerbate the financial strain on households in the lower-socioeconomic strata^11^.

RA exacerbates tremendous economic and social consequences in terms of lower quality of life, higher medical costs, productivity loss, and early retirement^12,13^. Prior studies have revealed that increasing out-of-pocket spending can lead to financial catastrophe for households, especially from lower-middle-income countries (LMICs)^14–16^. However, there is a lack of studies conducted in the Indian setting that estimate or report catastrophic health expenditure (CHE) among RA patients. Given this context, data on out-of-pocket expenditure (OOPE) and CHE among RA patients as well as the proportion of families experiencing CHE, are needed to estimate the economic burden. The purpose of the study is to estimate the burden of CHE and its major determinants of RA patients and their households and to give an insight into the economic impact of RA in Tamil Nadu, India.

## Methods

We conducted a cross-sectional descriptive and analytical hospital-based single-centre study at a tertiary care private multispecialty hospital in Tamil Nadu, India. The study centre is a 100-bed medical facility that specializes in rheumatology, cardiology, and nephrology. The study centre served 8789 new patients and 19,650 existing patients in the year 2022 as inpatients/outpatients. It also offers dedicated cardiac casualty services, intensive care, and comprehensive physiotherapy and diagnostic services for both outpatient and inpatient care. The study comprised 320 RA patients who visited the outpatient clinic from April to October 2022 and satisfied the inclusion criteria. Sample size estimation was performed a priori based on the prevalence of different severities in RA with 15% relative precision, 95% confidence interval (CI), 10% non-response, and a design effect of 1. Using systematic non-random sampling, every third RA patient who met the inclusion criteria was included in the study. RA Patients over 18 years who have had at least one follow-up visit following diagnosis are considered eligible for participation in the study. Patients with RA who also had other rheumatic or autoimmune disorders were excluded.

A pretested paper-based structured interview schedule available in English and Tamil was used to collect information from the study participants. Along with personal and household income details, socio-demographic data, such as age, gender, location, education level, occupation, household size, number of earning members in the household, as well as health care utilization data on the number of visits to the hospital, hospitalizations, medicine costs, physician fee, lab test charges were collected. We also collected non-medical costs such as food, accommodation, and transportation for the patient and the caregiver. Further data on insurance availability, premiums paid, and reimbursement were also collected. The rigorous training of interviewers on all aspects of the study helped to maintain quality data collection. The collected data were entered into Microsoft Excel, version 2019^17^, and the quality of the data entry was ensured by having a second-person review. Out of the total 320 participants interviewed, five were not included in the calculation of CHE due to their refusal to disclose income details.

### Data analysis

Demographic and baseline patient characteristics were reported using frequencies, percentages, mean, median, standard deviation (SD), and interquartile range (IQR). Household annual income, health expenditure for treating RA and OOPE were expressed as mean (95% CI) or median (IQR). All costs are reported in Indian rupee (₹) and US dollar ($), with a conversion factor of 1 US$ = ₹82.4^18^.

CHE is defined as health expenditure that exceeds a certain threshold of a patient's/family’s ability to pay, and medical expenses over and above the threshold are considered a significant financial burden for households. Based on previous research^14,19–21^, we defined CHE as spending more than 10% of the total household annual income on medical expenses due to RA and estimated the proportion of CHE. Also, as sensitivity analysis, we reported CHE considering 5% and 20% of the total household annual income scenarios.

Pearson’s Chi-square test for association was used to identify statistical significance, and multivariable logistic regression analyses were performed to identify major determinants associated with CHE. We also used concentration indices and the Lorenz curve to report the inequality in household annual income and CHE among the study participants. The concentration index measures inequality in the distribution of a variable of interest. The concentration index value ranges from − 1 to 1, with − 1 indicating that the outcome is concentrated in the lower socioeconomic group. A value of 1 suggests that the result is concentrated among the higher socioeconomic group. The Lorenz curve, which depicts the cumulative percentage of the outcome versus the variable of interest, is a graphical representation of the concentration index. The degree of inequality in the distribution of the outcome is represented by the gap between the 45-degree line of equality and the Lorenz curve. Violin plots are used to visualize the distribution and the density of multiple variables. A p-value < 0.05 was considered statistically significant. All the analyses were performed using Stata V.17^22^.

### Ethical approval

This study was carried out following the Helsinki Declaration's ethical guidelines. The ethical committees of the ICMR-National Institute of Epidemiology (NIE/IHEC/202101-01) and the CMMH (CMMHEC/21/09) approved the study protocol. To take part in the study, all participants provided informed consent.

## Results

### General characteristics of study participants

The majority of the study participants were females (88.1%) with mean age (SD) of 55.57 ± 12.29 years. Almost 93 per cent of participants were from urban areas, and 89.4 per cent were literate. The patient's household size ranged from 1 to 12, with a median (IQR) of 4 (2), and nearly 77 per cent of the households had one to three earning members in their family. Less than 3 per cent of the participants have smoking and alcohol consumption habits. As per body mass index (BMI), 34.7 per cent were overweight, 28.1 per cent were normal, 27.2 per cent were obese, and the rest (3.4%) were underweight. The mean disease duration among the participants was 8.65 ± 7.47 years with a median (IQR) of 7 (33), and 85 per cent of the study participants had moderate to severe disease activity [disease activity score (DAS)28 > 3.2]. At the same time, nearly 33 per cent reported a severe functional disability [Health Assessment Questionnaire (HAQ) > 1.5]. Only 8.1 per cent of participants said having health insurance, and 51.4 per cent of patients were assessed to have CHE. Table 1 depicts the general characteristics of the 320 RA patients examined in this study.

**Table 1:** General characteristics of study participants and frequency of facing CHEs.

Variables	Categories	Numbers (n = 320)	Frequency of facing CHEs (n = 315)		Sig
			CHE-No (N = 153)	CHE-Yes (N = 162)	
Gender	Male	38 (11.9)	21 (13.7)	17 (10.5)	0.379
	Female	282 (88.1)	132 (86.3)	145 (89.5)	
Age	18–30 years	8 (2.5)	7 (4.5)	1 (0.6)	0.148
	30–50 years	103 (32.2)	48 (31.4)	53 (32.7)	
	50–70 years	174 (54.4)	83 (54.3)	88 (54.3)	
	More than 70 years	35 (10.9)	15 (9.8)	20 (12.4)	
Place of residence	Urban	299 (93.4)	148 (97.3)	147 (90.7)	0.029
	Rural	21 (6.6)	5 (2.3)	15 (9.3)	
Household size	1–2	103 (32.2)	37 (24.2)	66 (40.7)	0.000
	3–5	168 (52.5)	94 (61.4)	72 (44.4)	
	More than 5	37 (11.5)	21 (13.7)	15 (9.3)	
	Not reported	12 (3.8)	1 (0.7)	9 (5.6)	
Education status	Literate	286 (89.4)	6 (3.9)	25 (15.4)	0.003
	Illiterate	32 (10.0)	146 (95.4)	136 (84.0)	
	Not reported	2 (0.6)	1 (0.7)	1 (0.6)	
Employment status	Working	66 (20.6)	42 (27.5)	24 (14.8)	0.009
	Not working	245 (76.6)	105 (68.6)	135 (83.3)	
	Not reported	9 (2.8)	6 (3.9)	3 (1.9)	
Household Earning members	None	13 (4.1)	1 (0.7)	12 (7.4)	0.000
	1–3	270 (84.3)	133 (87.7)	136 (84.0)	
	4 and above	21 (6.6)	17 (10.2)	3 (1.8)	
	Not reported	16 (5.0)	2 (1.4)	11 (6.8)	
Household Income quartile^#^	First	124 (39.4)	11 (7.2)	113 (69.7)	0.000
	Second	51 (16.1)	23 (15.0)	28 (17.3)	
	Third	68 (21.6)	51 (33.3)	17 (10.5)	
	Fourth	72 (22.9)	68 (44.5)	4 (2.5)	
Smoking	Yes	9 (2.8)	3 (2.0)	6 (3.7)	0.353
Drinking	Yes	8 (2.5)	2 (1.3)	6 (3.7)	0.177
BMI	Underweight (< 18.5)	11 (3.4)	6 (3.9)	5 (3.1)	0.084
	Normal (18.5 to < 25)	90 (28.1)	46 (30.1)	41 (25.3)	
	Overweight (< 25 to 30)	111 (34.7)	60 (39.2)	50 (30.9)	
	Obesity (> 30)	87 (27.2)	31 (20.3)	56 (34.5)	
	Not reported	21 (6.6)	10 (6.5)	10 (6.2)	
Insurance availability	Yes	26 (8.1)	15 (9.8)	11 (6.8)	0.331
Carpal tunnel	Yes	100 (31.3)	43 (28.1)	55 (34.0)	0.263
	No	220 (68.7)	110 (71.9)	107 (66.0)	
Tarsal tunnel	Yes	121 (37.8)	57 (37.2)	63 (38.9)	0.765
	No	199 (62.2)	96 (62.8)	99 (61.1)	
Disease duration	Less than 1 year	44 (13.8)	26 (17.0)	18 (11.1)	0.188
	1–5 years	81 (25.3)	40 (26.1)	40 (24.7)	
	5–10 years	86 (26.9)	33 (21.6)	51 (31.5)	
	10–20 years	88 (27.5)	46 (30.1)	41 (25.3)	
	More than 20 years	21 (6.6)	8 (5.2)	12 (7.4)	
Rheumatoid factor	Positive	237 (74.0)	111 (72.6)	122 (75.3)	0.278
	Negative	69 (21.6)	38 (24.8)	31 (19.1)	
	Not reported	14 (4.4)	4 (2.6)	9 (5.6)	
Anti-cyclic citrullinated peptide	Positive	188 (58.8)	90 (58.8)	95 (58.6)	0.988
	Negative	80 (25.0)	39 (25.5)	41 (25.3)	
	Not reported	52 (16.2)	24 (15.7)	26 (16.1)	
Functional status	Mild (HAQ < 1)	159 (49.7)	84 (54.9)	73 (45.1)	
	Moderate (HAQ 1 to 1.5)	56 (17.5)	26 (17.0)	30 (18.5)	0.191
	Severe (HAQ > 1.5)	105 (32.8)	43 (28.1)	59 (36.4)	
Disease activity	Remission (DAS < 2.6)	21 (6.6)	13 (8.5)	8 (4.9)	0.020
	Low (DAS 2.6 to < 3.2)	27 (8.4)	17 (11.1)	9 (5.6)	
	Moderate (DAS 3.2 to < 5.1)	141 (44.1)	73 (47.7)	67 (41.4)	
	Severe (DAS > 5.1)	131 (40.9)	50 (32.7)	78 (48.1)	

Figures in parathesis are percentage to row total #n = 315.

### Income and health expenditure pattern among RA patients

The mean (95% CI) household annual income of the participants was ₹710,492 (540,155 to 880,828) with a median (IQR) of ₹360,000 (420,000) [$4369 ($5097)]. The mean (95% CI) annual health expenditure for treating RA was estimated at ₹44,700 (41,710 to 47,690) with a median (IQR) of ₹39,210 (25,500) [$476 ($310)]. The corresponding mean (95% CI) and median (IQR) OOPE among RA patients per household were ₹40,698 (38,249 to 43,148) [$494 ($464 to $524)] and ₹36,450 (23,070) [$442 ($280)] respectively (Supplementary Table 1).

### Catastrophic health expenditure and its major determinants among RA patients

Households experiencing CHE owing to RA were 51.4% (n = 162). The burden was shown to be higher in some subpopulations, including urban persons (90.7%), females (89.5%), families with 1–3 earning members (84.0%), patients with lower education levels (84.0%), and elderly (> 50 years) (66.7%). Similarly, CHE is more prevalent among obese persons (34.5%), patients with more than five years of illness (64.3%), Rheumatoid factor (RF) positive (75.3%), anti-citrullinated protein antibody (anti-CCP) positive (58.6%), and people with severe RA (48.1%). The presence of CHE is more evident among patients in the first (69.7%) and second (17.3%) income quartiles and patients with mild functional disability (45.1%) (Table 1).

**Figure 1:**
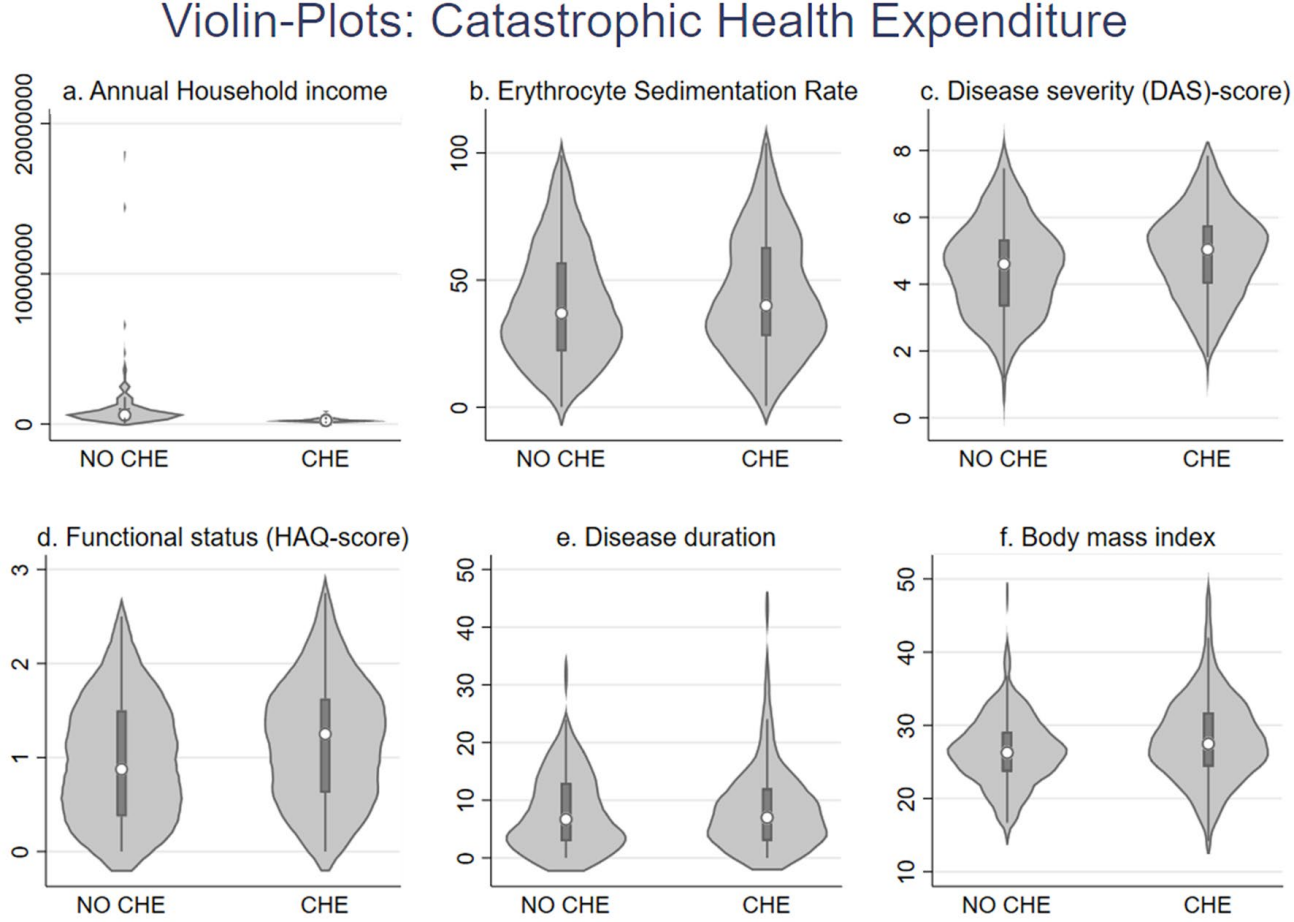
Violin plots for catastrophic health expenditure.

The violin plots (Fig. 1) show a significant difference in the distribution of annual household income, erythrocyte sedimentation rate (ESR), disease severity, functional status, disease duration, and BMI for CHE and no CHE categories. The median (IQR) of ESR is 40 (35), DAS28 is 5.04 (1.74), HAQ score is 1.25 (1), and BMI 27.47 (7.3) are high among people experiencing CHE. Similarly, a higher disease duration is found among patients who experience CHE with a median (IQR) of 7 (9). Similarly, the median (IQR) household annual income of those who experience CHE is ₹240,000 (120,000), much lower than that of non-CHE people [₹600,000 (600,000)].

The number of earning members and income quartiles were the primary predictors of CHE in RA patients; families with no earning member and one to three earning members had an odds ratio (OR) (95% CI) of 68 (6.29–735.3) and 5.79 (1.66–20.23), respectively (*p* < 0.001). Patients in the first income quadrant had a greater likelihood of suffering CHE with an OR (95% CI) of 174 (53.48, 570.18) (p < 0.001). Other major drivers were unemployment [OR 2.25 (1.28–3.95)], participants from urban area [OR 0.33 (0.12–0.93)], households with less than five members [OR 0.43 (0.26–0.71)], all with p < 0.001 (Table 2).

**Table 2:** Association between facing CHEs and household characteristics from logistic regression.

Variables	Categories	Crude odds ratio (95% CI)	Adjusted odds ratio (95% CI)
Place of residence	Rural	Referent	
	Urban	0.33 (0.12,0.93)*	0.14 (0.01, 1.12)
Household size	1–2	Referent	
	3–5	0.43 (0.26,0.71)*	0.49 (0.22, 1.11)
	More than 5	0.40 (0.18, 0.87)	0.48 (0.14, 1.63)
	Not reported	5.04 (0.61, 41.40)	1.84 (0.02, 169.10)
Education status	Literate	Referent	
	Illiterate	4.47 (1.78,11.24)*	1.47 (0.36, 5.95)
	Not reported	1.07 (0.66,17.33)	0.17 (0.00, 7.14)
Employment status	Working	Referent	
	Not working	2.25 (1.28, 3.95)*	1.25 (0.48, 3.28)
	Not reported	0.88 (0.20, 3.82)	0.01 (0.00, 0.24)
Household earning members	4 and above	Referent	
	None	68.0 (6.29, 735.3)*	37.24 (1.09, 1277.26)
	1 to 3	5.79 (1.66, 20.23)*	4.65 (0.59, 36.34)
	Not Reported	31.17 (4.46, 217.60)*	5.80 (0.17, 194.30)
Household income quintile	Fourth	Referent	
	First	174 (53.48, 570.18)*	**218.74 (55.29, 865.45)**
	Second	20.69 (6.55, 65.32)*	**21.49 (5.95, 77.58)**
	Third	5.67 (1.79, 17.86)*	**5.20 (1.47, 18.35)**
BMI	Healthy weight (18.5 to < 25)	Referent	
	Underweight (< 18.5)	0.93 (0.27, 3.29)	2.98 (0.42, 20.99)
	Overweight (< 25 to 30)	0.93 (0.53, 1.65)	1.22 (0.49, 3.08)
	Obesity (> 30)	2.03 (1.10, 3.72)*	3.15 (1.13, 8.78)
	Not reported	1.12 (0.42, 2.97)	2.44 (0.43, 13.80)
Disease activity	Remission (DAS < 2.6)	Referent	
	Low (DAS 2.6 to < 3.2)	0.86 (0.26,2.84)	1.09 (0.12, 9.83)
	Moderate (DAS 3.2 to < 5.1)	1.49 (0.58, 3.82)	0.76 (0.13, 4.42)
	Severe (DAS > 5.1)	2.54 (0.98, 6.55)	1.97 (0.34, 11.43)

*p < 0.05, Bold in adjusted are significant at p < 0.05.

When the potential risk factors for CHE in RA patients were examined, significant differences were found in family size, education level, job status, number of earning members, household income quartiles, BMI, and disease activity (Table 1). We used multivariate logistic regression to determine the effects of the above said factors on the likelihood that participants will have CHE. The logistic regression model was statistically significant (*p* < 0.001), χ^2^ = 221.77, and explained 51% (Nagelkerke R^2^) of the variance in CHE. Sensitivity analyses found that 78.4% (n = 247) and 22.5% (n = 71) of the households faced CHE, using 5% and 20% of household annual income thresholds for calculating CHE.

### Concentration index for income inequality

**Figure 2:**
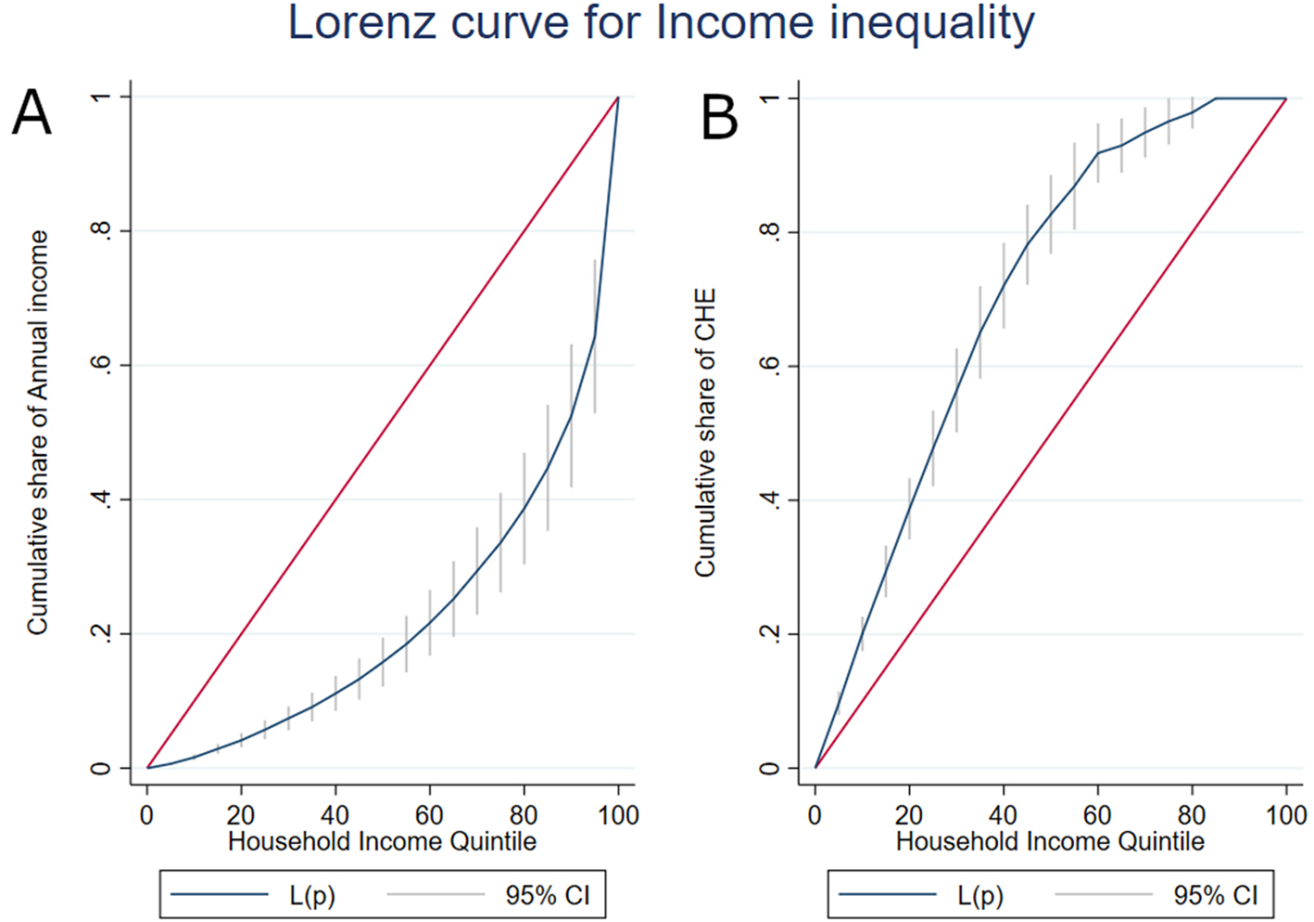
Lorenz curve for income inequality among the study participants.

The concentration index for annual household income with a score of 0.56 (*p* < 0.001) indicates that income is concentrated among the upper quintile (4th and 5th) participants. The Lorenz curve (Fig. 2a) shows that participants in the 5th quintile contributed roughly 40% of total income. On the contrary, the concentration index and Lorenz curve for CHE with a score of − 0.41 (*p* < 0.05) show that CHE is concentrated among participants in the lower income groups (Fig. 2b). Almost 80% of the CHE is contributed by low-income and lower-middle-income patients.

## Discussion

The study aimed to estimate the burden of CHE and OOPE and their major determining factors on households with RA patients in Tamil Nadu, India. The study found that the majority of the RA patients were female, literate, and from urban areas. The mean disease duration was 8.65 years, with 85% of the participants having moderate-to-severe disease activity. The mean household annual income was ₹710,492, concentrated mostly among the higher-income patients, with a mean annual health expenditure for treating RA estimated at ₹44,700.

The average OOPE per household was ₹40,698. More than half of the households experienced CHE, with a higher burden seen in rural areas and among those with lower income and education levels. The findings of this study provide vital information on the economic impact of RA on households of RA patients. The CHE burden is high among illiterates, females, and aged urban participants. The hospital's location in an urban region possibly contributed to the over-representation of urban patients in our analysis. There is also a substantial variation in the distribution of income, illness severity, functional status, disease duration, and BMI between the CHE and no CHE groups. CHE is more prevalent in individuals with more severe illnesses, longer disease duration and low median income. Both disease severity and disease duration result in a longer and more intensive treatment cycle, which results in higher treatment costs and CHE. The burden of CHE was substantially influenced by the family size, number of earning members, education, occupational status, and income.

The proportion of CHE was higher among lower-income households. Our findings showed that as the income of the household increased, the proportion of CHE decreased. Low-income families often choose not to seek healthcare to avoid financial hardships^23^ and OOPE caused by high healthcare costs and inadequate insurance coverage^10,15,24^. The lower insurance coverage rate among the participants and higher healthcare costs for RA likely contributed to an increased proportion of CHE due to the increased cost of treating RA incurred as OOPE. Households with no earning member and unemployed patients had a higher OR for having CHE than their counterparts. It is plausible that when the total household’s income is low, it becomes more vulnerable to financial difficulties^23,24^.

The estimated mean disease duration in our study is high, and studies have found that greater treatment costs are related to longer disease duration^25^. A study conducted in India in 2006 reported a cost burden of ₹49,142 in 2022 after adjusting for inflation^26^. Other studies conducted globally have reported average annual treatment costs for RA patients, with a 2001 study in the United States estimating $9519^27^ and a Scottish study estimating £4444^28^. Similarly, the average annual total cost for patients with high disease activity was $13,303 more than for patients in remission^25^. Previous research has shown that the incidence of CHE is about six times higher in the low-income group^19^. Financial protection schemes remain ineffective with the continued rise in health expenditure, which continues to contribute to CHE^23^; lower insurance coverage is a concern, as it will exacerbate CHE among RA patients^19^.

The study revealed that CHE is a major concern for RA patients and their families. Most of the RA drugs are expensive and given the average disease duration of 8.62 years, the requirement for longer medication adds to the overall treatment expenditures. Additionally, RA can have a considerable negative influence on daily activities and functioning, thereby increasing the cost of healthcare. The financial burden endured by RA patients can also be influenced by limited insurance coverage or insufficient reimbursement policies, which can lead to greater OOPE and CHE. The government of India has already implemented several schemes, such as Ayushman Bharat^29^ and Rashtriya Swasthya Bima Yojana^30^, to reduce OOPE and CHE by providing financial support to manage healthcare costs and ensuring health insurance availability. Additionally, government subsidies and regulating the price of essential medicines through Jan Aushadhi to reduce the financial burden on patients who pay out of pocket.

This study provides the CHE burden in RA patients and highlights the measures taken for providing improved access to quality healthcare services and financial protection for RA patients in India. There are several limitations to consider when interpreting the findings. The study design was cross-sectional, private hospital-based, and single-centre, limiting the generalizability of the results to other regions and populations in India. Additionally, the relatively small representation of patients from rural areas in the sample further restricts the ability to make comprehensive generalizations regarding patients from rural areas. Thus, the study's findings should be evaluated in the context of a single facility study, and they may not apply to the entire country. Information on the source and amount of borrowing and selling of assets, which may have influenced household spending patterns, was not analysed due to data restrictions. Recall bias may have also impacted the estimation of direct and indirect costs and OOPE. Although participants were reluctant to share income information, multiple alternative questions were asked to address this issue. Despite these limitations, the study provides valuable insights into the economic impact of RA on patients and their households in terms of CHE and OOPE in India. Further research is needed to assess the economic impact of RA in other regions of India. In the context of India, implementing universal health coverage (UHC) would indeed be a valuable policy intervention. It would help address the gaps in healthcare access and financial risk faced by individuals, including those with conditions like RA. By striving for UHC, India can work towards achieving equitable healthcare access, reducing OOPE, and improving health outcomes for its population^31^.

## Conclusion

The overwhelming majority of RA-related health costs are borne by patients, which they pay out of pocket, resulting in a CHE burden for more than half of them. Higher treatment costs along with improper health insurance coverage resulted in a higher OOPE and CHE among RA patients. The results underscore the need for comprehensive approaches to strengthening public health policies along with financial risk protection and quality care in India.

### Supplementary Information

Supplementary Information.

## Data Availability

The datasets used and/or analysed during the current study are available from the corresponding author on reasonable request.
