# Supplementary material for: Household catastrophic health expenditures for rheumatoid arthritis: a single centre study from South India
Source: Sci Rep. 2023 Sep 16;13:15385. doi: 10.1038/s41598-023-42623-y (PMC10505146; doi:10.1038/s41598-023-42623-y)
Supplement: Supplementary file 1 — Supplementary Information. [file 41598_2023_42623_MOESM1_ESM.pdf]

Online Supplementary Material

Of the manuscript: Bagepally et al., Household Catastrophic Health Expenditures for Rheumatoid Arthritis: A Single Centre study from South India

Contents

Supplementary table 1: Estimated cost components 2

CHEERS 2022 Checklist 3

STROBE Statement—Checklist 5

Supplementary table 1: Estimated cost components

Variable	Mean	Std. Err.	95% Conf interval	
Household Annual income	710492	86550	540155	880828
Direct medical cost	40698	1245	38249	43148
Direct non-medical cost	4363	829	2730	5995
Total Direct cost	44603	1516	41620	47586
Drug cost	31786.7	1196.4	29432.6	34140.7
Lab test cost	6468	198	6078	6858
Physician fee	1949	62	1828	2070
Transport cost	3271	706	1882	4660
Food cost	443	62	321	564

CHEERS 2022 Checklist

Topic	No.	Item	Location where item is reported
	1	Identify the study as an economic evaluation and specify the interventions being compared.	NA
	2	Provide a structured summary that highlights context, key methods, results, and alternative analyses.	2
**Introduction**			
Background and objectives	3	Give the context for the study, the study question, and its practical relevance for decision making in policy or practice.	3
**Methods**			
Health economic analysis plan	4	Indicate whether a health economic analysis plan was developed and where available.	NA
Study population	5	Describe characteristics of the study population (such as age range, demographics, socioeconomic, or clinical characteristics).	4
Setting and location	6	Provide relevant contextual information that may influence findings.	4
Comparators	7	Describe the interventions or strategies being compared and why chosen.	NA
Perspective	8	State the perspective(s) adopted by the study and why chosen.	4
Time horizon	9	State the time horizon for the study and why appropriate.	4
Discount rate	10	Report the discount rate(s) and reason chosen.	NA
Selection of outcomes	11	Describe what outcomes were used as the measure(s) of benefit(s) and harm(s).	5
Measurement of outcomes	12	Describe how outcomes used to capture benefit(s) and harm(s) were measured.	5
Valuation of outcomes	13	Describe the population and methods used to measure and value outcomes.	5
Measurement and valuation of resources and costs	14	Describe how costs were valued.	5
Currency, price date, and conversion	15	Report the dates of the estimated resource quantities and unit costs, plus the currency and year of conversion.	4
Rationale and description of model	16	If modelling is used, describe in detail and why used. Report if the model is publicly available and where it can be accessed.	NA
Analytics and assumptions	17	Describe any methods for analysing or statistically transforming data, any extrapolation methods, and approaches for validating any model used.	NA
Characterising heterogeneity	18	Describe any methods used for estimating how the results of the study vary for subgroups.	5
Characterising distributional effects	19	Describe how impacts are distributed across different individuals or adjustments made to reflect priority populations.	5
Characterising uncertainty	20	Describe methods to characterise any sources of uncertainty in the analysis.	NA
Approach to engagement with patients and others affected by the study	21	Describe any approaches to engage patients or service recipients, the general public, communities, or stakeholders (such as clinicians or payers) in the design of the study.	5
**Results**			
Study parameters	22	Report all analytic inputs (such as values, ranges, references) including uncertainty or distributional assumptions.	5-7
Summary of main results	23	Report the mean values for the main categories of costs and outcomes of interest and summarise them in the most appropriate overall measure.	5-6
Effect of uncertainty	24	Describe how uncertainty about analytic judgments, inputs, or projections affect findings. Report the effect of choice of discount rate and time horizon, if applicable.	7
Effect of engagement with patients and others affected by the study	25	Report on any difference patient/service recipient, general public, community, or stakeholder involvement made to the approach or findings of the study	NA

Topic	No.	Item	Location where item is reported
**Discussion**			
Study findings, limitations, generalisability, and current knowledge	26	Report key findings, limitations, ethical or equity considerations not captured, and how these could affect patients, policy, or practice.	7-8
**Other relevant information**			
Source of funding	27	Describe how the study was funded and any role of the funder in the identification, design, conduct, and reporting of the analysis	1
Conflicts of interest	28	Report authors conflicts of interest according to journal or International Committee of Medical Journal Editors requirements.	11

From: Husereau D, Drummond M, Augustovski F, et al. Consolidated Health Economic Evaluation Reporting Standards 2022 (CHEERS 2022) Explanation and Elaboration: A Report of the ISPOR CHEERS II Good Practices Task Force. Value Health 2022;25. doi:10.1016/j.jval.2021.10.008

STROBE Statement—Checklist

	Item No	Recommendation	Page No
**Title and abstract**	1	(*a*) Indicate the study's design with a commonly used term in the title or the abstract	1,2
		(*b*) Provide in the abstract an informative and balanced summary of what was done and what was found	2
**Introduction**			
Background/rationale	2	Explain the scientific background and rationale for the investigation being reported	3
Objectives	3	State specific objectives, including any prespecified hypotheses	3
**Methods**			
Study design	4	Present key elements of study design early in the paper	4
Setting	5	Describe the setting, locations, and relevant dates, including periods of recruitment, exposure, follow-up, and data collection	4
Participants	6	(*a*) Give the eligibility criteria, and the sources and methods of selection of participants	4
Variables	7	Clearly define all outcomes, exposures, predictors, potential confounders, and effect modifiers. Give diagnostic criteria, if applicable	4-5
Data sources/ measurement	8*	For each variable of interest, give sources of data and details of methods of assessment (measurement). Describe comparability of assessment methods if there is more than one group	*5*
Bias	9	Describe any efforts to address potential sources of bias	NA
Study size	10	Explain how the study size was arrived at	4
Quantitative variables	11	Explain how quantitative variables were handled in the analyses. If applicable, describe which groupings were chosen and why	5
Statistical methods	12	(*a*) Describe all statistical methods, including those used to control for confounding	5
		(*b*) Describe any methods used to examine subgroups and interactions	5
		(*c*) Explain how missing data were addressed	4
		(*d*) If applicable, describe analytical methods taking account of sampling strategy	NA
		(*e*) Describe any sensitivity analyses	5
**Results**			
Participants	13*	(a) Report numbers of individuals at each stage of study—eg numbers potentially eligible, examined for eligibility, confirmed eligible, included in the study, completing follow-up, and analysed	5
		(b) Give reasons for non-participation at each stage	NA
		(c) Consider use of a flow diagram	
Descriptive data	14*	(a) Give characteristics of study participants (eg demographic, clinical, social) and information on exposures and potential confounders	5
		(b) Indicate number of participants with missing data for each variable of interest	NA
Outcome data	15*	Report numbers of outcome events or summary measures	5-7
Main results	16	(*a*) Give unadjusted estimates and, if applicable, confounder-adjusted estimates and their precision (eg, 95% confidence interval). Make clear which confounders were adjusted for and why they were included	5-7
		(*b*) Report category boundaries when continuous variables were categorized	5-6
		(*c*) If relevant, consider translating estimates of relative risk into absolute risk for a meaningful time period	5-6
Other analyses	17	Report other analyses done—eg analyses of subgroups and interactions, and sensitivity analyses	6
**Discussion**			
Key results	18	Summarise key results with reference to study objectives	7
Limitations	19	Discuss limitations of the study, taking into account sources of potential bias or imprecision. Discuss both direction and magnitude of any potential bias	9
Interpretation	20	Give a cautious overall interpretation of results considering objectives, limitations, multiplicity of analyses, results from similar studies, and other relevant evidence	7-8
Generalisability	21	Discuss the generalisability (external validity) of the study results	8
**Other information**			
Funding	22	Give the source of funding and the role of the funders for the present study and, if applicable, for the original study on which the present article is based	1

*Give information separately for exposed and unexposed groups.

The STROBE checklist is best used in conjunction with this article (freely available on the Web sites of PLoS Medicine at http://www.plosmedicine.org/, Annals of Internal Medicine at http://www.annals.org/, and Epidemiology at http://www.epidem.com/). Information on the STROBE Initiative is available at www.strobe-statement.org.
